# Comparison between Digital Casts and Cone Beam Computed Tomography for Measuring Maxillary Transverse Dimensions in Patients with Impacted Canines

**DOI:** 10.3390/children9020278

**Published:** 2022-02-17

**Authors:** Elena Martinez Madero, Jaime García Montarelo, Grace Stefany Aguayo, Conchita Martin

**Affiliations:** 1 Department of Orthodontics, Faculty of Odontology, Complutense University of Madrid, 28040 Madrid, Spain; jaime.garcia.montarelo@gmail.com (J.G.M.); elena_mm21@hotmail.com (G.S.A.); conchitamartin@odon.ucm.es (C.M.); 2 BIOCRAN (Craniofacial Biology: Orthodontics and Dentofacial Orthopedics) Research Group, Complutense University of Madrid, 28040 Madrid, Spain

**Keywords:** included canines, canine impaction, maxillary transverse dimension, CBCT transversal measurements, dental cast maxillary measurements, intermolar width, interpremolar width, Walaridge

## Abstract

Cone beam computed tomography (CBTCT) scans (*n* = 45) and digital dental casts (*n* = 45) were both used to measure the maxillary transverse dimensions in patients with impacted maxillary canines. The objectives were to explore the associations of these dimensions with the impaction and patient characteristics, and to compare the measurements between these techniques. The maxillary width was measured on scans and casts at the Walaridge, and the intermolar width and interpremolar width levels were measured at the first and second premolars (measured from the buccal grooves and the palatal cuspids, and the palatal and lingual amelocemental junctions). Two examiners independently compared the measurements between the control quadrants (without impaction) and the case quadrants (with impaction) in patients with unilateral impactions, and between the unilateral and bilateral impaction groups. The interclass correlation coefficient (ICC) was calculated to assess the interexaminer reliability and paired or independent Student’s *t*-tests and ANOVAs were used for comparisons. The ICCs were 0.887 and 0.919, globally, for the measurements on the CBCT scans and casts, respectively, which indicates the excellent interexaminer reliability. On the CBCT scans, statistically significant differences were found between the case and control quadrants in the transverse measurements at the lingual level on the upper first molars, and at the WALA ridge level on the upper second premolars (*p* < 0.05) in the unilateral impaction group. Significant differences were found between the case quadrants in the unilateral versus the bilateral groups at the WALA ridge on the second premolars in casts (*p* < 0.05), and at the lingual point on the first molars on the CBCT scans (*p* < 0.05). No statistically significant differences in the transverse measurements were observed between the impacted buccal and palatal canines on either the casts or CBCT scans. To the best of our knowledge, this is the first study to compare the transverse measurements between digital models and CBCT scans.

## 1. Introduction

The maxillary canines are the second most frequently impacted teeth after the third molars [1]. The etiology of their impaction is associated with local (high canine position in the maxillary arch and maxillary transverse dimension; and the agenesis, or microdontia, of the lateral incisors) and genetic factors [2,3,4]. Buccal canine displacement is most often associated with anterior maxillary transverse (dental and skeletal) deficiency and palatal displacement, with small or missing lateral incisors, which is consistent with the guidance theory [5].

Various authors have reported that patients with canine impactions have a maxillary transverse deficiency in the anterior portion of the dental arch, and that premaxillary skeletal deficiency is frequently associated with the buccal impaction of the maxillary canines [3,6]. It has also been observed that maxillary transverse excess can be associated with their palatal impaction [7], while another study found a narrower and longer maxillary arch in patients with palatal versus buccal impacted canines [5]. In contrast, Langberg concludes that the maxillary arch width is not a primary contributory factor in the genesis of palatal displaced canines (PDCs) [8], and Saiar et al. assert that the maxillary intercanine alveolar arch width is not a good predictor of PDCs [9]. Likewise, Yan et al. found that PDCs are largely associated with small or missing lateral incisors, which is in line with the guidance theory [10].

No consensus has been established on the best approach to maxillary transverse deficiency measurement [11]. Digital dental casts have been used for maxillary transverse measurements in patients with impacted canines by most authors [9,12,13,14], and cone beam computed tomography (CBCT) scans by others [15,16,17,18]. Hong et al. measured the maxillary basal bone and the interdental widths at the maxillary first molars and the first and second premolars on the axial and coronal CBCT sections [15], and Arboleda recorded measurements on images at four levels: at the first molar basal and alveolar widths, and at the first premolar basal and alveolar widths [16]. Cacciatore, Ghaffar, and Kim used digital dental casts to measure the maxillary dentoalveolar width, which is defined as the distance between the mesiobuccal cusp tips of the first molars [7,13,19], whereas Bizarro considered the intercanine and intermolar widths at the cusp and gingival levels [12]. In their study, Saiar et al. measured the maxillary intermolar width as the distance between the central grooves on the permanent maxillary first molars [9].

There has been little high-quality research on the maxillary transverse deficiencies and the results have been controversial, with some studies reporting that the arch width prediction indices obtained from the dental cast measurements are undependable. Measurement on CBCT images has been described as a more reliable diagnostic approach, but further validation is required to confirm its accuracy. No study has addressed the maxillary morphology using both digital dental casts and CBCTs.

In this study, digital dental casts and CBCT scans were both used to measure the maxillary dental arch transverse dimensions in patients with impacted canines. The study objectives were to explore the associations of these dimensions with the impaction and patient characteristics, and to compare the measurements between these techniques, with the aim of contributing to the development of standardized procedures for these measurements.

## 2. Materials and Methods

### 2.1. Subjects and Study Design

This is a transversal analytical study. The study sample was recruited from among the patients attending the Orthodontics Department Clinic at the Complutense University of Madrid (UCM) from 2005 through to 2021. Th study inclusion criteria were: (1) Aged over 11 years and cervical vertebral maturation (a CVM less than 4) [20]; (2) The presence of mixed or permanent dentition; (3) A sagittal discrepancy <3 mm with no need for extraction (nonextraction case); (4) A vertical overbite >3 mm, but <6 mm; and (5) A transverse maxillomandibular discrepancy <4 mm (nonsurgical case). The study exclusion criteria were: (1) A history of systematic disease and/or medication affecting the tooth movement or the bone metabolism; (2) Orofacial malformation syndrome; (3) A history of treatment with orthodontic/orthopedic appliances; (4) A defect in the X-ray or casts that could affect the measurements; and (5) An ankylosed canine (i.e., no movement after three months of treatment). The sample size estimation was based on a previous study by Cacciatore et al. (2018) [19], in which the mean difference between the affected and unaffected sides was 0.32 mm, with a SD of 0.44 mm. Setting an alpha significance level of 0.05 and a beta significance level of 0.10, for a power of 90%, to detect the intragroup differences, we concluded that 23 patients were needed in each group. A total of 50 patients were finally enrolled in order to cover the possible dropouts.

### 2.2. Methodology of Measurements 

For the measurements, the transverse width of the maxillary arch was divided between the control quadrant (without impaction) and the case quadrant (with impaction) in the patients with unilateral impactions, whereas both hemiarches were considered case quadrants in the patients with bilateral impactions. The total transverse widths were also compared between the unilateral and bilateral impactions, and between the buccal and palatal displacement. All the measurements were independently performed by two examiners (E.M. and J.G.).

For the measurements in the digital casts and CBCT scans, a modified method based on a combination of other studies by Hong et al. and Cacciatori et al. was used [15,19].

**Figure 1:**
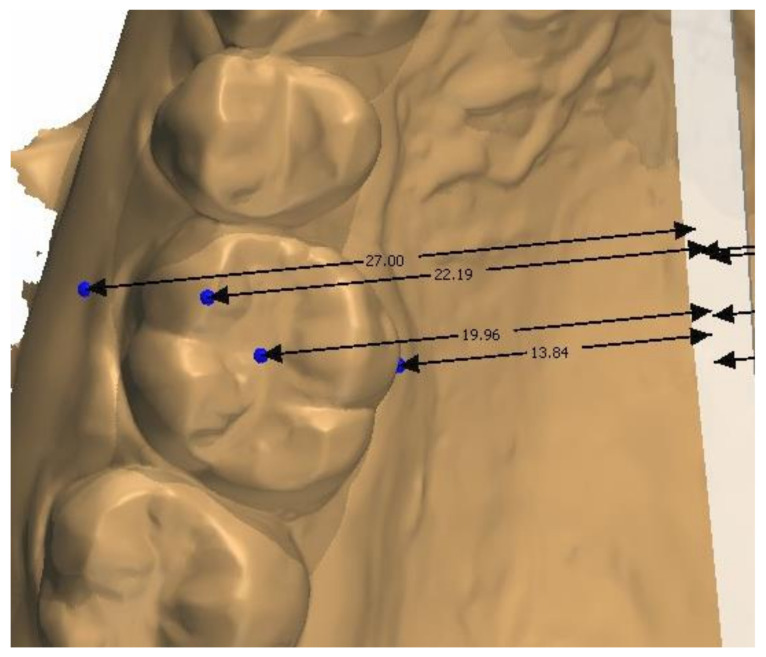
Points on first molars on digital casts.

**Figure 2:**
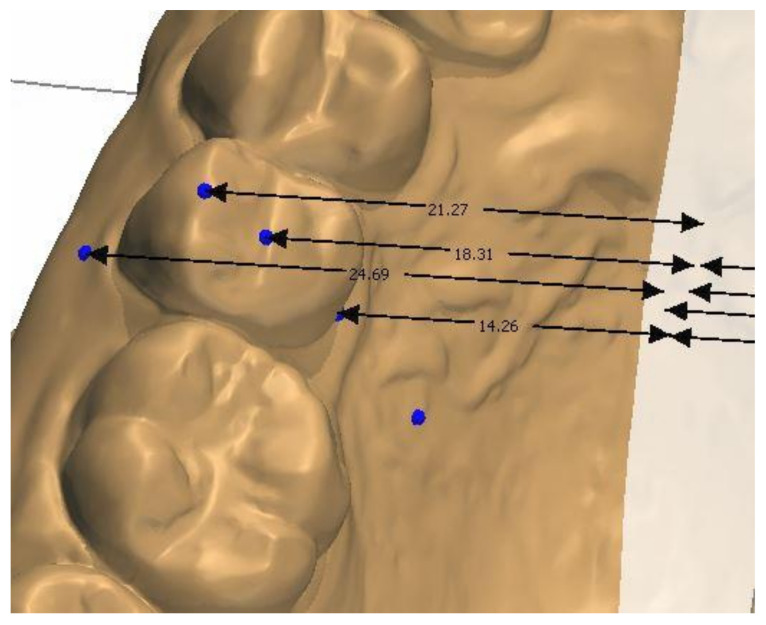
Points on second premolars on digital casts.

**Figure 3:**
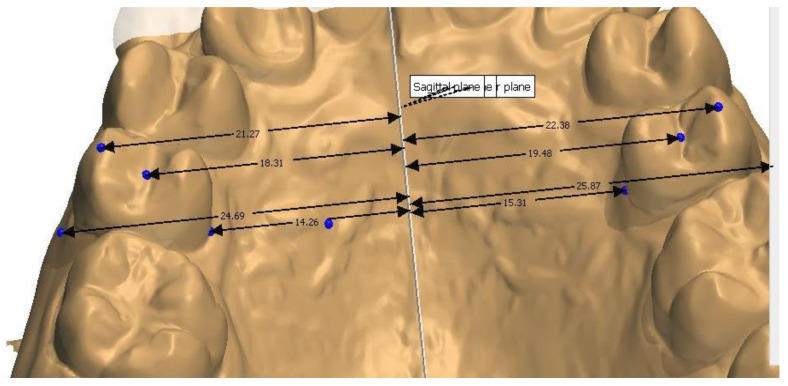
Distances from second premolar to medial sagittal plane.

**Figure 4:**
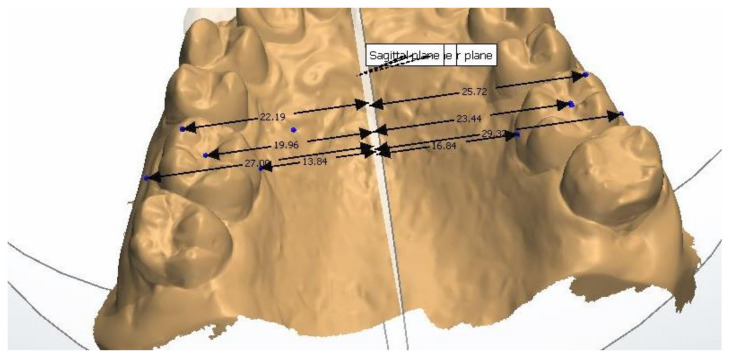
Distances from first molar to medial sagittal plane.

The study model casts were scanned using a 3D scanner (3Shape D700, Copenhagen, Denmark), with a reported accuracy of <20 microns (www.3shape.com, last accessed on 10 February 2022), which imported the 3D data into the OrthoAnalyzer software package (3Shape Systems, Inc. Copenhagen, Denmark). The authors used the alginate, Orthoprint (Zhermarck^®^, Rovigo, Italy), to make the impressions of the patients, as well as Type III plaster (Orthoguix plaster, Protechno^®^, Girona, Spain). The measurements were made from four points on the first molars and the first and second premolars: from the gingival level of the mesiopalatal cuspid, and from the central groove, the mesiobuccal cuspid, and the Walaridge point. The distances were measured from each point to the medial sagittal plane (i.e., perpendicular to the occlusal plane that crosses the palatine raphe (the posterior and medial points of the raphe)). The final measurements were the distances from the gingival level to the medial sagittal plane, from the central groove to the medial sagittal plane, from the mesiobuccal cuspid (buccal cuspid in premolars) to the medial sagittal plane, and from the Walaridge to the medial sagittal plane (Figure 1, Figure 2, Figure 3 and Figure 4).

**Figure 5:**
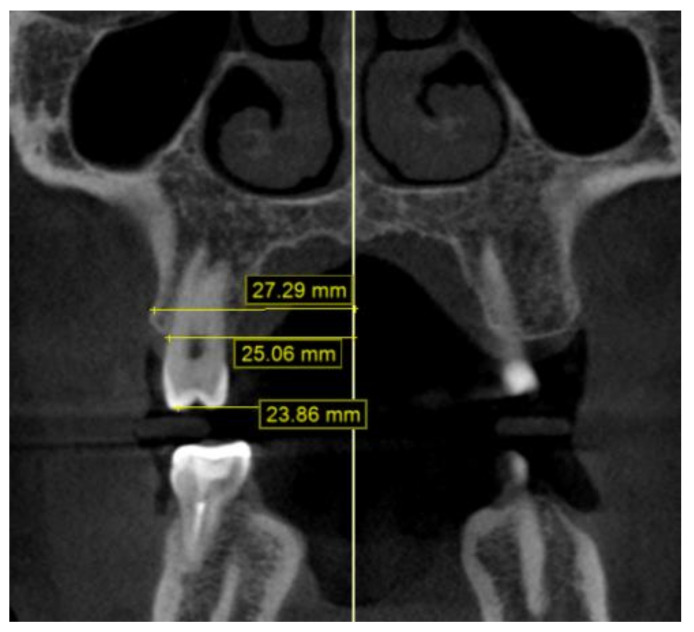
Distances from second premolar to medial sagittal plane on CBCT scan: Walaridge, gingival buccal level, and buccal cuspid.

**Figure 6:**
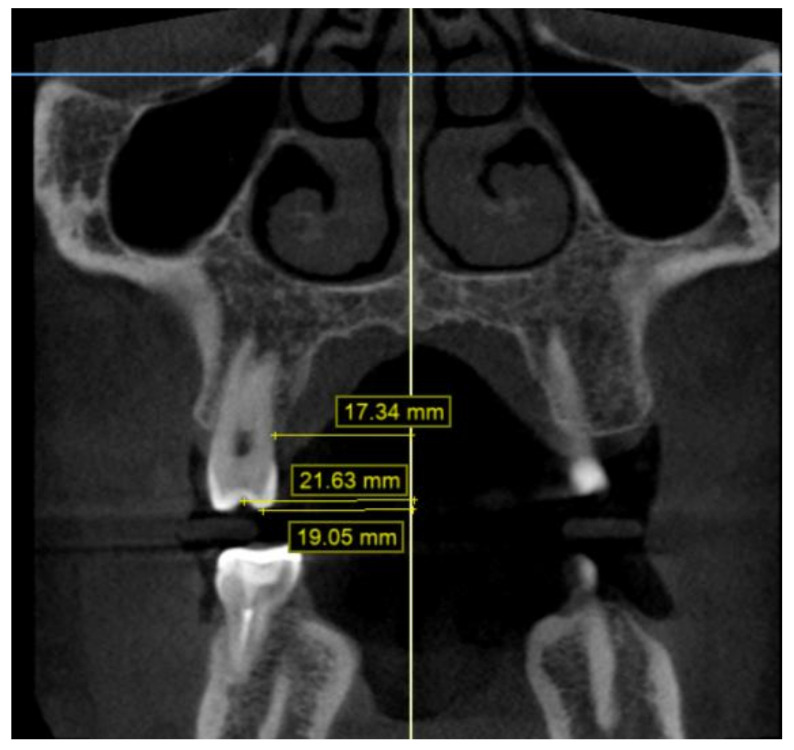
Distances from second premolar to medial sagittal plane on CBCT scan: groove, lingual gingival, and palatal cuspid.

**Figure 7:**
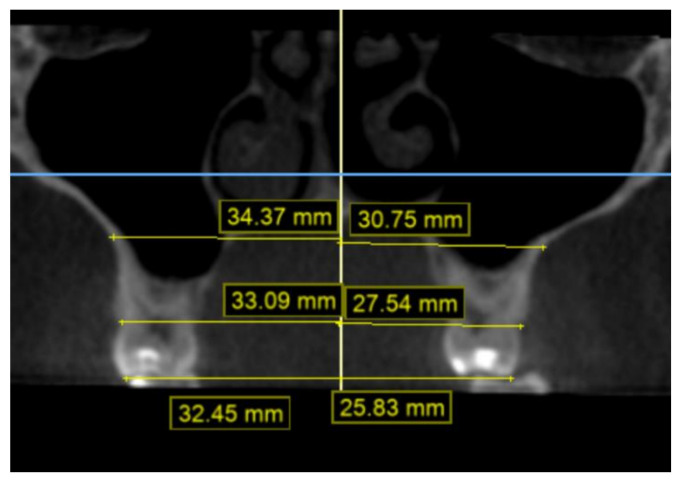
Distances from first molar to median sagittal plane on CBCT scan: Walaridge, groove, and J point.

The CBCT data were obtained using the i-CAT^®^ Cone Beam 3D Imaging System (Imaging Sciences International, Inc., Hatfield, PA, USA), with the patients in an upright position, and always using the same machine and parameters: a 17-cm × 23-cm field of view, 120 kV, 5 mA (pulsed mode), and a 0.3-mm voxel size. The measurements were made from six points on the first molars and the first and second premolars: the palatal cuspid, the buccal cuspid, the lingual gingival, the buccal gingival (amelocemental junction), the central groove, and the Walaridge. The distances were measured from each point to the medial sagittal plane (Figure 5 and Figure 6). The skeletal transverse distance was also measured as the distance between the J point and the sagittal medial plane (Figure 7).

### 2.3. Statistical Analysis

Means and standard deviations were calculated for each measurement. The interclass correlation coefficient (ICC) was used to assess the interexaminer agreement. After checking the normality of the data distribution with the Levene’s test, Student’s *t*-tests and analyses of variance (ANOVAs) were performed to analyze the relationships between the study variables, as specified in the table footnotes. SPSS version 25.0 (IBM Corp, Armonk, NY, USA) was used for the data analyses, which considered *p* < 0.05 as significant.

## 3. Results

### 3.1. Demographic Data

**Figure 8:**
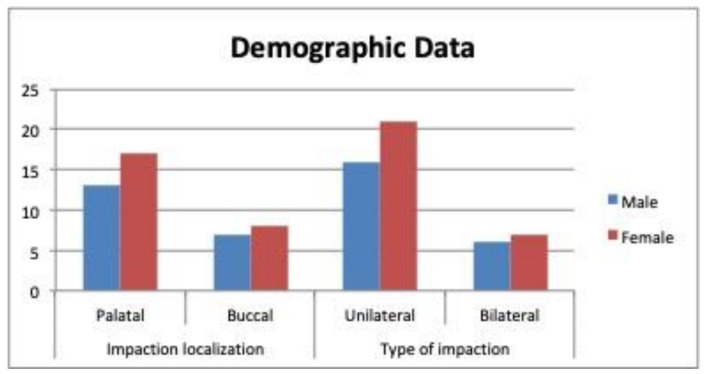
Patient characteristics: sex, palatal/buccal localizations of the impactions, and their unilateral or bilateral type.

Out of the 50 patients enrolled in the study, 5 were excluded for the poor quality of the X-rays or the casts, which left a final study sample of 45 patients (25 females and 20 males), with a total of 63 impacted maxillary canines. The mean (± SD) age was 15.42 ± 3.42 years (Table 1), and all of the participants had mixed or permanent dentition and cervical vertebral maturation at Stages 4 or 5. No significant differences in the study variables were observed between the sexes, or as a function of the patient age, the palatal/buccal displacement, or the unilateral/bilateral impaction (Table 1) (Figure 8). The ICC index for the interexaminer agreement was 0.919 for the cast measurements, and 0.887 for the CBCT measurements, which are, i.e., almost perfect correlations (Table 2).

### 3.2. Transverse Width

**Figure 9:**
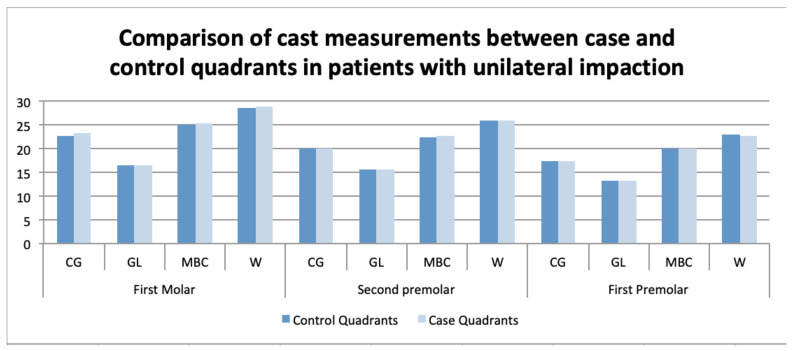
Comparison of cast measurements between the case and control quadrants in patients with unilateral impactions.

**Figure 10:**
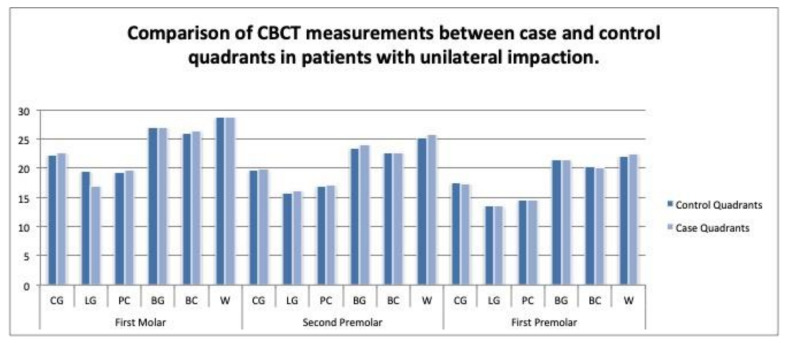
Comparison of CBCT scan measurements between the case and control quadrants in patients with unilateral impactions.

Unilateral impactions: In the digital models, no significant differences in the transverse widths were found between the case and control quadrants in the unilateral cases. On the CBCT scans, the intermolar width at the lingual gingival level (mean ± SD of 17.01 ± 1.96 mm/SD = 1.96; *p* < 0.001), and the interpremolar width at the WALA ridge level, were significantly narrower in the case versus the control quadrants (Table 3 and Table 4) and (Figure 9 and Figure 10).

**Figure 11:**
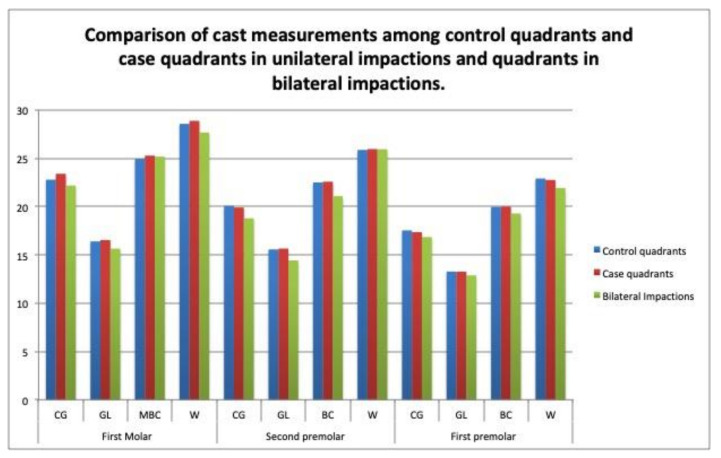
Comparison of cast measurements among the control quadrants and the case quadrants in the unilateral impactions, and the quadrants in the bilateral impactions.

Bilateral impactions: In the digital models, the interpremolar width at the buccal cuspid level on the second premolars was narrower in the patients with bilateral versus unilateral impactions (21.07 mm/SD = 1.58; *p* = 0.025). The Bonferroni comparisons showed statistically significant differences between the quadrants in the bilateral group and the case quadrants in the unilateral group (*p* = 0.027 *), with a mean difference of 1.59 mm (95% CI, 0.14–3.04) (Table 5), (Figure 11).

**Figure 12:**
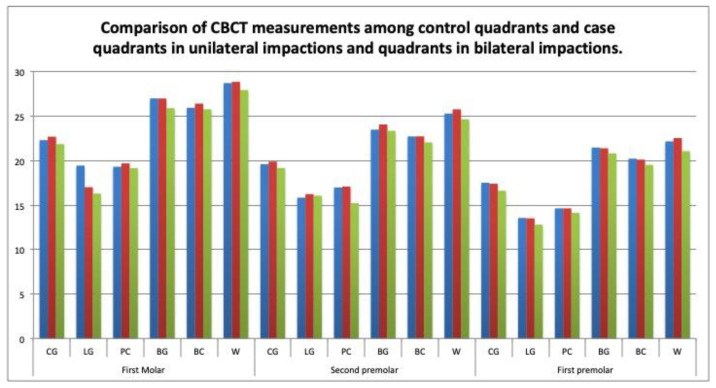
Comparison of CBCT measurements among control quadrants and case quadrants in unilateral impactions, and quadrants in bilateral impactions.

On the CBCT scans, a narrower intermolar width was found at the lingual gingival level (difference of 16.29 ± 1.15 mm; *p* < 0.001) and the Walaridge level (21.07 ± 1.45 mm; *p* = 0.05) on the first premolars in the bilateral versus unilateral impaction cases. The Bonferroni comparisons revealed statistically significant differences in the intermolar width at the gingival level (*p* < 0.001), and in the interpremolar width at the Walaridge level (difference of 1.46 mm (95%CI, 0.01 to 2.92; *p* = 0.047 *)) between the quadrants in the bilateral impaction group, and the case quadrants in the unilateral group. Significant differences were also found in the intermolar distance at the gingival level (mean difference of 3.04 mm (95% CI 1.73 to 4.36; *p* < 0.001)) between the quadrants in the bilateral impaction group and the case quadrants in the unilateral group, and in the intermolar distance at the gingival level between the case and control quadrants in the unilateral impactions (difference of 2.39 mm (95% CI, 1.29 to 3.48; *p* < 0.001)) (Table 6), (Figure 12).

**Figure 13:**
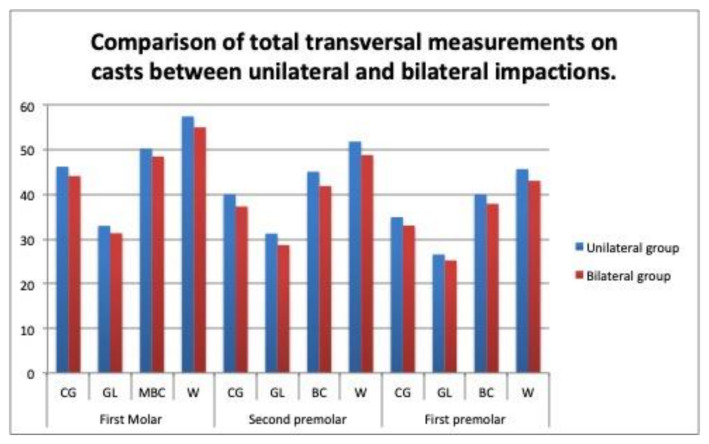
Comparison of total transversal measurements on casts between unilateral and bilateral impactions.

**Figure 14:**
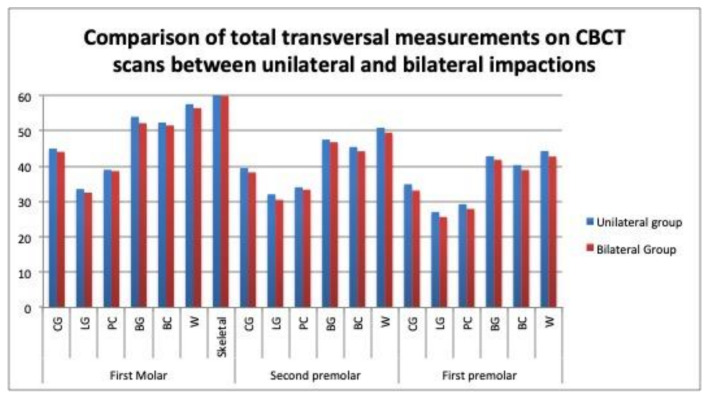
Comparison of total transversal measurements on CBCT scans between unilateral and bilateral impactions.

The total transverse widths were compared between the unilateral and bilateral cases, as they were measured on the dental casts and CBCT scans. On the casts, the measurements were significantly narrower in the bilateral cases at the central groove level (37.27 mm vs. 39.28 mm, *p* = 0.028), at the lingual gingival point (28.62 mm vs.31.21 mm, *p* = 0.041), and at the buccal cuspid (41.85 mm vs. 45.07 mm, *p* = 0.018) on the second premolars, and at the Walaridge level on the first molars (Table 7), (Figure 13). On the CBCT scans, no significant differences in the measurements were found between the bilateral and unilateral impaction groups (Table 8), (Figure 14).

**Figure 15:**
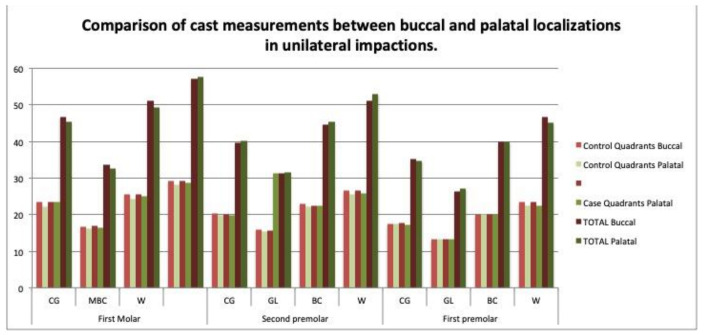
Comparison of cast measurements between buccal and palatal localizations in unilateral impactions.

**Figure 16:**
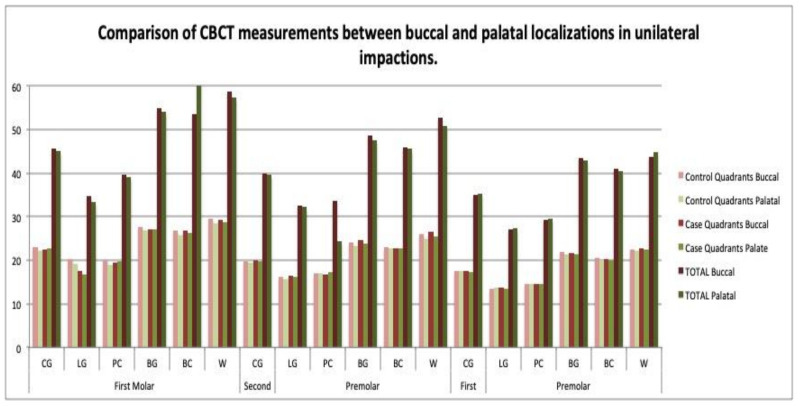
Comparison of CBCT measurements between buccal and palatal localizations in unilateral impactions.

No relationship was observed between the maxillary transverse measurements and the buccal or palatal localization of the impaction on the casts or CBCT scans, neither in the unilateral nor the bilateral cases (Table 9 and Table 10) (Figure 15 and Figure 16). No correlations were observed between the digital model and the CBCT scan measurements in almost any dimension (Table 11).

## 4. Discussion

In this study, both digital casts and CBCT scans were used to measure the maxillary transverse dimensions in patients with impacted canines, with the aim of evaluating the associations of the findings with the patient and impaction characteristics.

The profile of the study population was similar to that in previous investigations in terms of the buccal or palatal localization of the impactions, their unilateral or bilateral presence, and the sex distribution [8,10,12,21,22,23]. Thus, the impactions were more frequently palatal and unilateral, and there was a female:male ratio of 2:1, which is consistent with previous reports [8,15,19]. The maxillary dimensions are usually smaller in females than in males [16], and Hong et al. propose that the association between the impaction incidence and the female sex has a genetic origin [2,15] (Table 1) (Figure 8).

The ICC was 0.887 for all of the measurements on the CBCT scans, and almost perfect agreements were obtained at the lingual point levels on the first molars (0.961), the second premolars (0.943), and the first premolars (0.969). Other studies describe high interexaminer agreement (0.9) for the CBCT measurements [15,16,17], although one of these studies [17] used the cuspids on the first molars as the reference point, and their inclination or rotation could influence the results. The ICC was higher for the measurements on the casts (0.919) than for those on the CBCT scans in the present study, with the best reference point for the cast measurements being the lingual point on the first premolars (0.997), and the worst being the Walaridge on the upper first molars (0.6) (Table 2).

### 4.1. Transverse Dimensions in Case versus Control Quadrants in Unilateral Impactions

Numerous reports have been published on this issue, but the findings have been controversial. In the present study, the measurements in the digital casts showed no significant differences in the transverse dimensions between the case and control quadrants in the unilateral impaction cases. Most of the impacted canines were palatal, and it has been widely documented that there is no relationship between palatal impaction and maxillary transverse deficiency [8,9,10,12,13,15]. Although Cacciatori et al. observed a narrower and smaller palate in impacted canine cases versus controls, they also found no significant difference between the quadrants with impacted versus nonimpacted canines in the cases of unilateral impaction [19] (Table 3), (Figure 9).

On the CBCTS scans, a statistically significant difference between the case and control quadrants was found in the measurements at the lingual level on the upper first molars and at the Walaridge level on the upper second premolars (*p* < 0.05); however, the difference at the Walaridge level was not considered because of the low interexaminer agreement on this measurement. The width at the lingual point on the upper first molars was narrower in the case (with impacted canine) (17.01 mm) versus the control quadrants (19.44 mm) in the unilateral impaction cases. By contrast, Misrasmaeli at al. found no statistically significant difference in this measurement at the lingual level on the upper first molars [17]. The width at the lingual level has not been measured in previous CBCT studies [10,11,15,16] (Table 4), (Figure 10).

### 4.2. Transverse Dimensions in Unilateral versus Bilateral Impactions by Quadrant

On the casts, the measurements at the Walaridge point on the second premolars differed significantly between the case quadrants in the unilateral group and the quadrants in the bilateral group (*p* < 0.05). On the CBCT scans, the measurements at the lingual point on the first molars differed significantly between the case quadrants in the unilateral group and the quadrants in the bilateral group (*p* < 0.05). Our review of the literature found no study that compared the maxillary transverse measurements by quadrant between unilateral versus bilateral impactions. (Table 5 and Table 6), (Figure 11 and Figure 12).

### 4.3. Transverse Dimensions in Unilateral versus Bilateral Impactions by Arch

On the casts, the transverse measurements of the whole arch differed significantly between the patients with unilateral and bilateral impactions at the levels of the central groove, the lingual point, and the buccal cuspid on the second premolars (Table 7), (Figure 13). On the CBCT scans, no statistically significant differences were observed in any of the measurements (Table 8) (Figure 14). Mucedero and Arboleda also observed no significant differences in these dimensions between the unilateral and bilateral impactions [14,16].

### 4.4. Transverse Dimensions in Buccal versus Palatal Impactions

No significant differences in the transverse dimensions were observed between the buccal and palatal impactions on either the casts or the CBCT scans (Table 9 and Table 10) (Figure 15 and Figure 16). Arboleda et al. also found no differences in the transverse dimension at the first molar level [16]. By contrast, some authors describe a significantly narrower transverse width in the premolar region in cases of buccal impaction [10], and others have found an association between canine impaction and buccal displacement [14]. Therefore, the relationship between the palatal or buccal localization of impaction and the maxillary transverse dimensions is unclear [7].

### 4.5. Transverse Measurements on CBCT Scans versus Casts

Our review of the literature was unable to trace studies that compared the transverse measurements between the digital models and the CBCT scans. In the present study, four measurement points were selected that might, theoretically, provide more similar results between the two techniques. However, there were no correlations between these approaches in the dimensions obtained at these points. In the cases of unilateral impaction, statistically significant differences were found in the measurements at the central groove, the lingual point, and the Walaridge levels. Hence, the measurements obtained at the selected points with one of these techniques are not comparable to those obtained by the other (Table 11).

### 4.6. Clinical Relevance

The transverse measurements made in the CBCT scans and digital models could be used in the diagnosis of patients with transverse malocclusions or included canines. However, it is important to consider that there is not an absolute correspondence between the two diagnostic methods. A differential diagnosis will indicate if expansion is needed and should focus on the dental or skeletal components, or both.

According to our results, the bilateral impaction cases may require maxillary expansion more frequently than the unilateral impaction cases, where asymmetrical expansion might be considered, and CBCT scans and digital models are valid tools for assessing this asymmetry.

**Limitations:**

- Taking impressions with alginate and the subsequent casting in plaster may lead to certain accuracy errors in the digital models. The use of the latest-generation intraoral scanner could possibly produce a more accurate result because of the lack of cumulative errors, as the results from the systematic review by Jedliński et al. suggest [24].
- It would be worthwhile to compare the results obtained with the results obtained from intraoral scans;
- The infrabony position of the impacted canine, especially the distance to the occlusal plane, has not been considered (only its palatal or buccal position). The transversal maxillary development may be affected by this factor.

## 5. Conclusions

In terms of our main objective, we can conclude that there is no relationship between the maxillary canine impaction and the arch width measured by the quadrants at the dental, dentoalveolar, or skeletal levels. The maxillary transverse width measurements on the CBCT scans are not correlated with those on the digital casts at the central groove, lingual point, or Walaridge levels.

The secondary findings from our study are as follows:

1. The demographic data confirm that impacted canines were more frequent in females versus males, and when the impaction was palatal versus buccal, and unilateral versus bilateral. These impaction characteristics were not related to the maxillary arch width;
2. There is no relationship between the maxillary arch width and the buccal or palatal displacement of the impacted canine;
3. In the bilateral impactions, the maxillary arch had a smaller transverse width at the dental level. This difference was statistically significant on the digital casts but not on the CBCT scans;
4. In the unilateral impactions, statistically significant differences in the measurements were observed between the case and control quadrants on both the digital casts and the CBCT scans.

## Figures and Tables

**Table 1:** Patient characteristics: sex, palatal/buccal localizations of the impactions, and their unilateral or bilateral type.

Gender	Impaction Localization		Type of Impaction		Age (Years)
	Palatal	Buccal	Unilateral	Bilateral	μ ± SD
Male	13	7	16	6	15.61 ± 3.42
Female	17	8	21	7	14.66 ± 4.20
TOTAL	30	15	34	11	
*p* value	0.504		0.554		0.806

**Table 2:** Interclass coefficients for interexaminer agreement.

Interclass Coefficients for Interexaminer Agreement	Median	Max	Min
Casts	0.919	0.997	0.54
CBCT scans	0.887	0.988	0.32

**Table 3:** Comparison of cast measurements between the case and control quadrants in patients with unilateral impactions.

Tooth	Parameter	Control Quadrants	Case Quadrants	*p* Value *
First Molar	CG	22.78	23.4	0.103
	GL	16.4	16.54	0.671
	MBC	24.94	25.28	0.343
	W	28.56	28.87	0.286
Second Premolar	CG	20.07	19.9	0.56
	GL	15.56	15.65	0.758
	MBC	22.5	22.57	0.798
	W	25.86	25.95	0.65
First Premolar	CG	17.53	17.35	0.506
	GL	13.27	13.26	0.935
	MBC	19.97	20	0.919
	W	22.9	22.75	0.56

Student’s *t*-test for paired samples. GL: gingival level; CG: central groove; MBC: mesiobuccal cuspid; W: Walaridge. * *p* < 0.05 as significant.

**Table 4:** Comparison of CBCT measurements between the case and control quadrants in patients with unilateral impactions.

Tooth	Parameter	Control Quadrants	Case Quadrants	*p* Value *
First Molar	CG	22.3	22.69	0.265
	LG	19.44	17.01	**0.001** *
	PC	19.31	19.69	0.281
	BG	27	26.99	0.956
	BC	25.94	26.41	0.162
	W	28.72	28.86	0.642
Second Premolar	CG	19.6	19.89	0.303
	LG	15.83	16.23	0.175
	PC	16.98	17.08	0.771
	BG	23.48	24.07	0.158
	BC	22.72	22.73	0.984
	W	25.29	25.77	**0.048** *
First Premolar	CG	17.51	17.4	0.724
	LG	13.55	13.51	0.882
	PC	14.63	14.63	0.99
	BG	21.47	21.39	0.852
	BC	20.22	20.11	0.746
	W	22.16	22.54	0.163

Student’s *t*-test for paired samples; the statistically significant differences are in bold. LG: lingual gingival; CG: central groove; PC: palatal cuspid; BG: buccal gingival; BC: buccal cuspid; and W: Walaridge. * Statistically significant *p* ≤ 0.05.

**Table 5:** Comparison of cast measurements among the control quadrants and case quadrants in unilateral impactions, and the quadrants in bilateral impactions.

Tooth	Parameter	Control Quadrants	Case Quadrants	Bilateral Impactions	*p* Value *
First Molar	CG	22.78	23.39	22.17	0. 145
	GL	16.39	16.53	15.64	0. 267
	MBC	24.94	25.27	25.17	0.456
	W	28.56	28.87	27.66	0.456
Second Premolar	CG	20.07	19.9	18.78	0.069
	GL	15.56	15.65	14.42	0.76
	BC	22.5	22.57	21.07	**0.025** *
	W	25.86	25.95	25.93	0.145
First Premolar	CG	17.53	17.35	16.84	0.487
	GL	13.27	13.26	12.87	0.716
	BC	19.97	20	19.28	0.373
	W	22.9	22.75	21.92	0.277

ANOVA; statistically significant difference (*p* ≤ 0.05) is in bold. GL: gingival level; CG: central groove; MBC: mesiobuccal cuspid; BC: buccal cuspid; W: Walaridge. * Statistically significant *p* ≤ 0.05.

**Table 6:** Comparison of CBCT measurements among control quadrants and case quadrants in unilateral impactions, and quadrants in bilateral impactions.

Tooth	Parameter	Control Quadrants	Case Quadrants	Bilateral Group	*p* Value *
First Molar	CG	22.3	22.69	21.86	0.231
	LG	19.44	17.01	16.29	**0.001** *
	PC	19.31	19.69	19.16	0.556
	BG	27	26.99	25.9	0.093
	BC	25.94	26.41	25.77	0.413
	W	28.72	28.86	27.94	0.375
Second Premolar	CG	19.6	19.89	19.17	0.427
	LG	15.83	16.23	32.05	0.208
	PC	16.98	17.08	15.22	0.914
	BG	23.48	24.07	23.35	0.587
	BC	22.72	22.73	22.05	0.399
	W	25.29	25.77	24.65	0.208
First Premolar	CG	17.51	17.4	16.61	0.204
	LG	13.55	13.51	12.79	0.384
	PC	14.63	14.63	14.12	0.691
	BG	21.47	21.39	20.82	0.539
	BC	20.22	20.11	19.52	0.558
	W	22.16	22.54	21.07	**0.051** *

ANOVA; statistically significant (*p* ≤ 0.05) and close-to-significant results are in bold. PC: palatal cuspid; BC: buccal cuspid; LG: lingual gingival; BG: buccal gingival (cementoenamel junction); CG: central groove; and W: Walaridge. * Statistically significant *p* ≤ 0.05.

**Table 7:** Comparison of total transversal measurements on casts between unilateral and bilateral impactions.

Tooth	Parameter	Unilateral Group	Bilateral Group	*n*	*p* Value *
First Molar	CG	46.18	44.1	2.08	0. 071
	GL	32.94	31.29	1.65	0. 120
	MBC	50.22	48.45	1.77	0.209
	W	57.44	54.99	2.45	**0.054** *
Second Premolar	CG	39.98	37.27	2.71	**0.028** *
	GL	31.21	28.65	2.59	**0.041** *
	BC	45.07	41.85	3.23	**0.018** *
	W	51.81	48.8	2.95	**0.060** *
First Premolar	CG	34.88	33	1.88	0.08
	GL	26.53	25.2	1.34	0.323
	BC	39.97	37.88	2.09	0.094
	W	45.65	43.02	2.63	0.128

ANOVA; statistically significant and close-to-significant results are in bold. CG: central groove; GL: gingival lingual; MBC: mesiobuccal cuspid; BC: buccal cuspid (premolars); and W: Walaridge. * Statistically significant *p* ≤ 0.05.

**Table 8:** Comparison of total transversal measurements on CBCT scans between unilateral and bilateral impactions.

Tooth	Parameter	Unilateral Group	Bilateral Group	*n*	*p* Value *
First Molar	CG	44.99	44.07	0.92	0.365
	LG	33.55	32.54	1	0.385
	PC	39	38.62	0.38	0.792
	BG	53.99	52.17	1.82	0.141
	BC	52.35	51.53	0.82	0.543
	W	57.58	56.44	1.14	0.497
	Skeletal	61.12	59.94	1.19	0.385
Second Premolar	CG	39.48	38.25	1.23	0.391
	LG	32.06	30.47	1.59	0.214
	PC	34.05	33.35	0.7	0.596
	BG	47.55	46.79	0.76	0.465
	BC	45.45	44.23	1.22	0.298
	W	50.9	49.49	1.41	0.307
First Premolar	CG	34.91	33.1	1.81	0.093
	LG	27.06	25.64	1.42	0.306
	PC	29.26	27.91	1.35	0.315
	BG	42.84	41.82	1.04	0.622
	BC	40.33	38.93	1.4	0.139
	W	44.31	42.74	1.57	0.315

ANOVA. PC: palatal cuspid; BC: buccal cuspid; LG lingual gingival; BG: buccal gingival; CG: central groove; and W: Walaridge. * Statistically significant *p* ≤ 0.05.

**Table 9:** Comparison of cast measurements between buccal and palatal localizations in unilateral impactions.

Tooth	Parameter	Control Quadrants			Case Quadrants			Total		
		Buccal	Palatal	*p* Value *	Buccal	Palatal	*p* Value *	Buccal	Palatal	*p* Value *
First Molar	CG	23.42	22.1	0.133	23.39	23.49	0. 953	46.82	45.53	0.35
	GL	16.75	16.03	0.235	16.86	16.47	0.644	33.62	32.56	0.389
	MBC	25.65	24.25	0.188	25.43	25.03	0. 616	51.09	49.29	0.229
	W	29.24	28.09	0.193	29.3	28.75	0.441	57.07	57.6	0.249
Second Premolar	CG	20.36	19.8	0.494	19.95	19.79	0.828	39.6	40.32	0.615
	GL	16	15.37	0.443	15.51	31.28	0.485	31.28	31.51	0.866
	BC	23.01	22.23	0.343	22.48	22.47	0.987	44.7	45.5	0.583
	W	26.54	25.48	0.2	26.58	25.73	0.3	51.21	53.12	0.23
First Premolar	CG	17.55	17.56	0.997	17.61	17.22	0.555	35.16	34.78	0.779
	GL	13.21	13.33	0.803	13.25	13.33	0.906	26.46	27.24	0.6
	BC	19.99	19.97	0.986	19.97	19.97	0.995	39.96	39.98	0.989
	W	23.37	22.5	0.414	23.34	22.5	0.307	46.7	45.2	0.32

* Student’s *t*-test for independent samples. CG: central groove; GL: gingival lingual; MBC: mesiobuccal cuspid; BC: buccal cuspid (premolars); and W: Walaridge. * Statistically significant *p* ≤ 0.05.

**Table 10:** Comparison of CBCT measurements between buccal and palatal localizations in unilateral impactions.

Tooth	Parameter	Control Quadrants			Case Quadrants			Total		
		Buccal	Palatal	*p* Value *	Buccal	Palate	*p* Value *	Buccal	Palatal	*p* Value *
First Molar	CG	23.02	22.04	0.135	22.5	22.75	0.761	45.53	44.79	0.556
	LG	20.23	19.15	0.063	17.52	16.81	0.422	34.83	33.07	0.166
	PC	20.09	19.02	0.102	19.42	19.78	0.646	39.51	38.81	0.566
	BG	27.74	26.72	0.134	27.05	26.95	0.912	54.8	53.68	0.422
	BC	26.82	25.61	0.08	26.72	26.29	0.631	53.55	51.991	0.244
	W	29.45	28.45	0.223	29.28	28.7	0.558	58.73	57.15	0.347
Second Premolar	CG	19.84	19.5	0.612	20.01	19.84	0.834	39.86	39.35	0.701
	LG	16.11	15.73	0.567	16.36	16.18	0.833	32.47	31.91	0.684
	PC	16.92	16.99	0.924	16.8	17.16	0.66	33.73	24.16	0.754
	BG	23.95	23.3	0.602	24.52	23.9	0.483	48.48	47.21	0.51
	BC	22.93	22.64	0.682	22.82	22.69	0.868	45.76	45.33	0.75
	W	26.06	25.01	0.15	26.57	25.49	0.091	52.63	50.51	0.176
First Premolar	CG	17.45	17.52	0.93	17.55	17.34	0.783	35.01	34.87	0.921
	LG	13.41	13.59	0.841	13.67	13.44	0.723	27.09	27.04	0.972
	PC	14.61	14.64	0.977	14.6	14.63	0.968	29.21	29.27	0.968
	BG	21.9	21.31	0.507	21.62	21.31	0.713	43.53	42.62	0.544
	BC	20.59	20.09	0.415	20.37	20.01	0.692	40.96	40.1	0.619
	W	22.5	22.04	0.368	22.77	22.46	0.757	43.76	44.5	0.673

Student’s *t*-test for independent samples. CG: central groove; GL: gingival lingual; MBC: mesiobuccal cuspid; BC: buccal cuspid (premolars), and W: Walaridge. * Statistically significant *p* ≤ 0.05.

**Table 11:** Comparison between digital cast and CBCT measurements.

Tooth	Parameter	Control Quadrants			Case Quadrants			Total		
		Cast	CBCT	*p* Value *	Cast	CBCT	*p* Value *	Cast	CBCT	*p* Value *
First Molar	CG	22.55	22.37	0.548	23.28	22.7	**0.041** *	45.83	45.07	**0.012** *
	GL	16.28	19.5	**0.0001** *	16.42	16.97	**0.049** *	32.7	33.56	**0.001** *
	W	28.37	28.74	**0.018** *	28.71	28.86	0.526	57.07	57.6	**0.018** *
Second Premolar	CG	19.93	19.64	0.336	19.63	19.91	0.232	39.56	39.54	0.96
	GL	15.53	15.86	0.251	15.61	16.27	**0.018** *	31.14	32.12	**0.001** *
	W	25.66	25.36	**0.001** *	25.8	25.81	0.937	51.45	51.18	0.306
First Premolar	CG	17.46	17.49	0.883	17.13	17.22	0.606	34.59	34.71	0.406
	GL	13.28	13.82	0.055	13.14	13.55	0.063	26.67	27.89	**0.001** *
	W	22.78	22.31	**0.026** *	22.42	22.54	0.699	45.3	44.38	0.164

Student’s *t*-test for paired samples; statistically significant results (*p* ≤ 0.05) are in bold. CG: central groove; LG: lingual gingival; and W: Walaridge. * Statistically significant *p* ≤ 0.05.

## Data Availability

Not applicable.
